# Module detection in complex networks using integer optimisation

**DOI:** 10.1186/1748-7188-5-36

**Published:** 2010-11-12

**Authors:** Gang Xu, Laura Bennett, Lazaros G Papageorgiou, Sophia Tsoka

**Affiliations:** 1 Centre for Process Systems Engineering, Department of Chemical Engineering, University College London, Torrington Place, London, WC1E 7JE, UK; 2 Centre for Bioinformatics, Department of Informatics, School of Natural and Mathematical Sciences, King's College London, Strand, London, WC2R 2LS, UK

## Abstract

**Background:**

The detection of *modules or community structure *is widely used to reveal the underlying properties of complex networks in biology, as well as physical and social sciences. Since the adoption of modularity as a measure of network topological properties, several methodologies for the discovery of community structure based on modularity maximisation have been developed. However, satisfactory partitions of large graphs with modest computational resources are particularly challenging due to the NP-hard nature of the related optimisation problem. Furthermore, it has been suggested that optimising the modularity metric can reach a resolution limit whereby the algorithm fails to detect smaller communities than a specific size in large networks.

**Results:**

We present a novel solution approach to identify community structure in large complex networks and address resolution limitations in module detection. The proposed algorithm employs modularity to express network community structure and it is based on mixed integer optimisation models. The solution procedure is extended through an iterative procedure to diminish effects that tend to agglomerate smaller modules (resolution limitations).

**Conclusions:**

A comprehensive comparative analysis of methodologies for module detection based on modularity maximisation shows that our approach outperforms previously reported methods. Furthermore, in contrast to previous reports, we propose a strategy to handle resolution limitations in modularity maximisation. Overall, we illustrate ways to improve existing methodologies for community structure identification so as to increase its efficiency and applicability.

## Background

Networks - i.e. groups of entities (*nodes *or *vertices*) pairs of which are linked through a form of common property (*edges *or *links*) - have formed an efficient representation framework for a variety of complex systems such as social groupings and internet connectivity [1]. The analysis of biological data in systems biology studies through the formalisms of network theory have received particular attention recently, due to the potential benefits that such methodologies can confer in mining the intricate relationships in metabolic networks [2-4], signaling pathways [5], gene regulatory networks [6] or other forms of protein interactions [7]. In general, the abstractions offered by graph theory representations (i) facilitate the analysis of network performance, (ii) provide a unifying framework for comparisons of features across different systems and (iii) assist the mathematical characterisation of system properties and dynamics.

Topological properties of networks are particularly important in revealing the organisational principles of nodes within the context of the entire system [8]. *Community structures *or *modules *are defined when a larger density of links exists within a specific part of the network than outside it [9]. Each of such modules can be regarded as a discrete entity whose function or properties are in some way separable from other modules. Modular structure underlies (i) the adaptability of a system to new conditions [10] and (ii) the robustness (or conversely the *vulnerability*) of the system to external attack or other form of change in topological features [11,12]. The analysis of pairwise or even longer-range relationships in networks can reveal how preferential attachment of new nodes influences community structure [13,14], giving rise to small-world or scale-free architectures [15].

In light of the above, the detection of modules and the analysis of community structure in networks has the potential to reveal the design principles of complex systems and provide important insights into how such systems are organised, how they evolve and how their components interact. For example, in biological networks, the analysis of enzyme connectedness may reveal participation in the same biological pathway, and module detection in protein interactions reflects protein function type or evolutionary properties [3,7,16]. Importantly, the characterisation of gene products with previously unknown functional properties through community detection has the potential to aid function assignment. Recent reviews have reported on the community detection problem in comprehensive manner [17,18].

The two major avenues to detect community structure have been graph partitioning [19,20] and hierarchical clustering methods [16,20-23]. Major disadvantage in the case of graph partitioning is the absence of a termination criterion in the bisection process, while in hierarchical clustering there is no clear indication of where the tree should be split to yield the optimal partitioning. Such shortcomings result in either sub-optimal partitioning or unsatisfactory implementations for large networks. In addition to graph partitioning and hierarchical methods, which are particularly suitable for standard partitioning (each node belongs to a single community), other methodologies exist to detect overlapping communities as nodes may belong to several communities, for example, the clique percolation method, [24].

An important breakthrough in the community detection problem has taken the form of a quantitative measure to express the quality of community presence, namely *modularity*. Network modularity is defined as the fraction of all edges that lie within communities minus the expected value of the same quantity in a graph in which the vertices have the same degrees but edges are placed randomly [25-27]. Usually, in our experience, network modularity values of around 0.4-0.8 indicate strong community presence. Use of the modularity metric has transformed the community structure identification problem into an optimisation task where community structures can be determined by maximising the network modularity through various optimisation techniques [25].

As modularity optimisation is NP-hard [28,29], efficient algorithms to find the maximum modularity values are unlikely to exist. Therefore, most approaches employ heuristics that aim at finding near-optimal solutions with modest computational cost. Crucial considerations in assessing the performance of modularity optimisation approaches are: (i) the scale and optimality handled by modularity optimisation methods and (ii) the resolution limit problem for small-size modules in large networks.

First, there seems to be a trade-off between network size and optimality achieved through modularity optimisation. Specifically, methods that guarantee global optimal solutions for modularity maximisation are able to operate only in small to medium-sized networks [30]. Divisive algorithms [25] were found to be prohibitively computationally expensive for large networks. On the other hand, methods that can be used on large networks, such as stochastic optimisation through simulated annealing [3,31] and extremal optimisation [32], may yield sub-optimal solutions and so may suffer poor performance. In our own work, we have previously reported a rigorous mixed integer quadratic programming (MIQP) formulation to optimise the modularity metric with a set of linear constraints and mixed binary/continuous optimisation variables [30]. Due to the convexity properties of the model, global optimal solutions are achieved through the standard branch-and-bound procedure with commercial optimisation solvers, but use of this optimisation framework is limited to small-medium scale networks due to NP-hardness.

Second, doubts have been raised over the use of modularity optimisation for community detection recently, due to the observation that such procedures can reach a resolution limit [33]. This effect essentially implies that modules smaller than a specific scale are not detected, as the optimisation process combines smaller communities into larger ones in order to achieve better modularity. Some remedial procedures have been suggested through re-optimising each module [33,34], tuning a resolution parameter [35], or implementing quantitative measures other than modularity [36].

Here, we aim to enhance the application of mathematical programming to community structure identification by: (i) developing an efficient methodology for module detection that is capable of handling large size networks and (ii) incorporating strategies for dealing systematically with the problem of a resolution limit in module detection through modularity optimisation approaches. Below, a two-stage solution approach for community identification using mathematical programming is described, the resolution limit in modularity optimisation is addressed via the introduction of an iterative procedure and the applicability of the proposed approaches is demonstrated through a number of network examples and comparisons with literature.

## Methods

**Figure 1:**
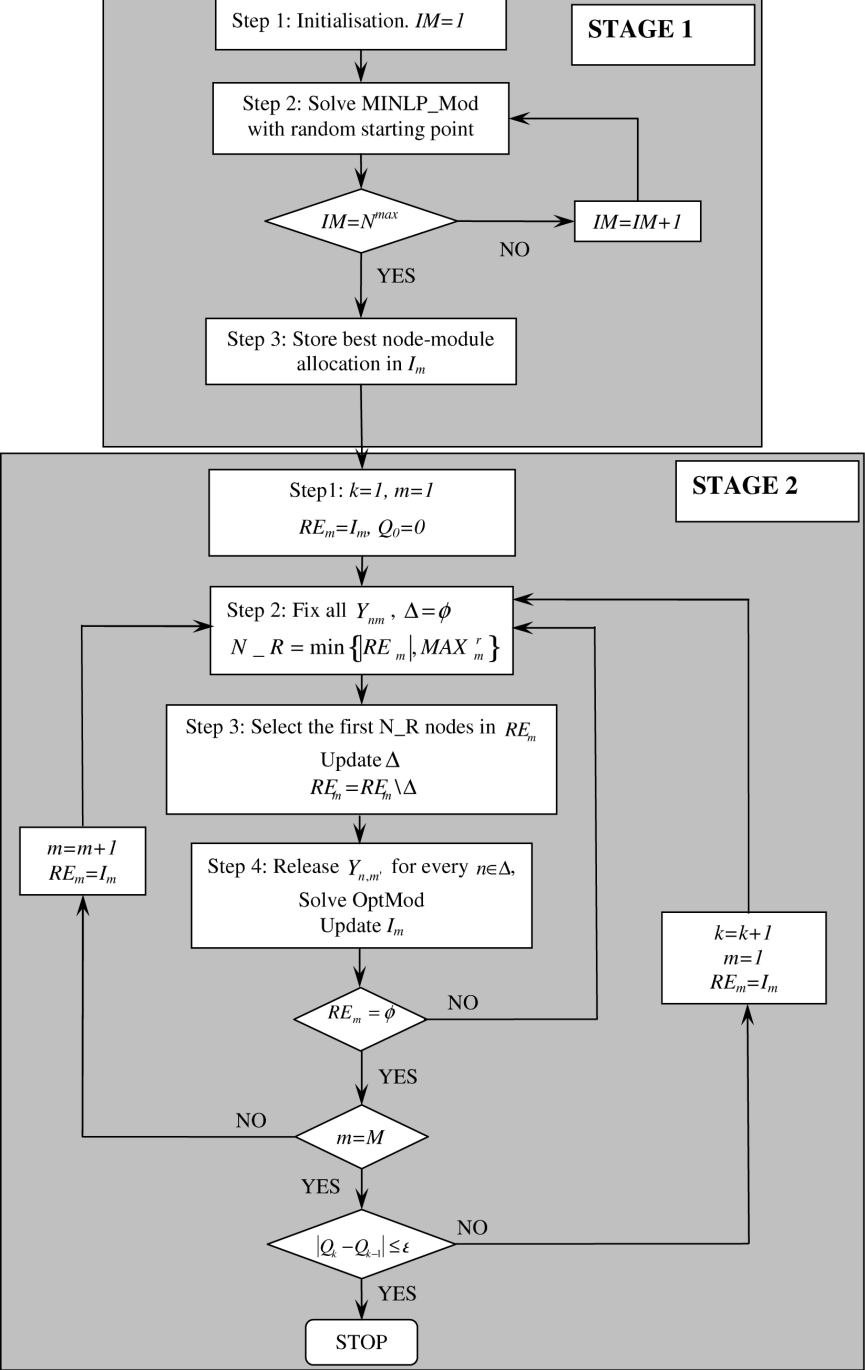
**Flowchart of the iMod algorithm for module detection**. For details on the solution procedure above, please refer to the text.

The solution approach presented in this paper is a two-stage, iterative modularity optimisation procedure, named iMod. First, a mixed integer nonlinear programming (MINLP) model (MINLP_Mod) is formulated to obtain a feasible solution efficiently. An initial partition with a good modularity value is selected from a set of MINLP solutions with random starting points. Second, the solution obtained in the first stage is improved through an iterative optimisation procedure employing a model that we have developed previously and was proven efficient in detecting communities in small to medium size networks through a global maximum of the modularity metric (OptMod, [30]). Overall, the iMod approach that combines the two aforementioned stages is intended to extend the use of mathematical programming methodologies to larger-size networks. A schematic representation of the iMod computational procedure, combining MINLP_Mod and OptMod, is shown in Figure 1.

### Stage 1: Initial network partition

Given a network with *N *nodes and *L *edges, the modularity metric, *Q*, of a network partitioned into *M *communities is represented as:

(1) $$Q=\sum_{m}\left[\frac{L_{m}}{L}-{\left(\frac{D_{m}}{2L}\right)}^{2}\right]$$

where *L*_*m *_denotes the number of links in module *m *and *D*_*m *_is the degree of all nodes in module *m*. The modularity metric, *Q*, measures the difference between the fraction of links within communities and the expected fraction values when links are allocated randomly [25,26]. The objective function employed here is the maximisation of the network modularity metric shown in equation (1).

First, each node is allocated to exactly one module:

(2) $$\begin{array}{cc} \sum_{m}Y_{nm}=1 & \forall n \end{array}$$

where *Y*_*nm *_is a binary variable taking the value of *1 *if node *n *is allocated to module *m*; *0 *otherwise.

As previously defined, *D*_*m *_is equal to the sum of the degrees of nodes allocated to module *m*:

(3) $$\begin{array}{cc} D_{m}=\sum_{n}d_{n}\cdot Y_{nm} & \forall m \end{array}$$

A link will be allocated to module *m *only when both nodes associated with it are also in module *m*. Therefore, the total number of links in module *m*, *L*_*m*_, is defined by the following nonlinear equality:

(4) $$\begin{array}{cc} L_{m}=\sum_{n}\sum_{\begin{array}{l} e>n\\ e\in CN_{n} \end{array}}Y_{nm}\cdot Y_{em} & \forall m \end{array}$$

where *CN*_*n *_is the set of nodes *e *connected to node *n*.

Overall, the resulting MINLP model (MINLP_Mod) for determining community structures based on the modularity metric maximisation is formulated as:

(5) $$\begin{array}{l} \text{Maximise}:Q=\sum_{m}\left[\frac{L_{m}}{L}-{\left(\frac{D_{m}}{2L}\right)}^{2}\right]\\ \text{Subject to: Constraints (2-4)} \end{array}$$

(6) $$\begin{array}{cc} L_{m},D_{m}\geq 0 & \forall m \end{array}$$

(7) $$Y_{nm}\in \left\{0,1\right\}$$

Since global optimality of non-convex MINLP models cannot be guaranteed, different initial solutions are tested and the partition with the largest value of *Q*, is chosen as the best division from the set of candidate solutions. MINLP_Mod is performed for a given number of runs, *N*^*max*^, from random initial points and the node-module allocation with the maximum modularity value is stored and denoted by set *I*_*m*_. Using *N*^*max *^= 100 provides a good representation of solution space.

### Stage 2: Iterative improvement of network partition

Having selected a node-module association with maximum modularity from the previous stage (*i.e*. *I*_*m*_), module allocation may be improved further through an iterative fixing and releasing scheme. The general idea is to solve a reduced MIQP formulation of modularity optimisation that was previously proposed in [28]. Most of the *Y*_*nm *_variables are fixed, which reduces the number of variables, thus resulting in a more tractable model. Sets of nodes are released in the sense that they are free to be re-allocated to a different module in subsequent executions of the MIQP model.

This stage initially adopts the node-module allocation obtained from stage 1 by fixing all the relevant *Y*_*nm *_binary variables in *I*_*m *_to the value of one. For the first module, the set of nodes in the module is denoted as *RE*_*m *_and the set of nodes to be released (or 'un-fixed') is denoted as Δ, where the size of Δ is *N_R*, a value chosen according to criteria described below. The reduced MIQP (OptMod) is solved (for details see [30]), with all nodes in Δ released and all other nodes fixed. *I*_*m *_is then updated with the solution from the reduced MIQP.

The above scheme is applied sequentially for remaining modules, which completes one round of the major improvement iteration, *k*, with network modularity value, *Q*_*k*_. The same strategy starts again, retaining the order of the modules, until no improvement of the modularity value is reported for two successive major iterations.

Comparing the single-level MIQP model, OptMod, to the reduced MIQP models as implemented here, the latter strategy involves fewer variables and constraints and can be terminated efficiently even in cases of larger size networks, as discussed in the Results and Discussion section. To justify why an iterative reduced MIQP is preferred over MINLPs, it should be mentioned that we achieved improved solutions by solving a series of reduced MIQP models, while no improvements have been observed when solving reduced MINLP models iteratively.

To avoid releasing too many nodes so that the reduced OptMod model is still difficult to solve, the maximum number of released nodes for module *m*, $MAX_{m}^{r}$, is set to:

(8) $$MAX_{m}^{r}=\frac{U}{Aver_{m}}$$

where *Aver*_*m *_denotes the average degree in module *m *without considering the inter-module links and *U *is a user-defined parameter. Here, we used a value of *U = 200 *which was shown to provide satisfactory results for all examples studied. As a result, the actual number of released nodes, *N_R*, will be the smaller value between *MAX*_*m*_^*r *^and the number of remaining nodes to be released in *RE*_*m *_(*i.e*. $N\_R=\min\{\left|RE_{m}\right|,MAX_{m}^{r}\}$).

In other words, if the number of nodes in module *m *is greater than $MAX_{m}^{r}$, the first *N_R *nodes, Δ, in module *m *will be released and the reduced MIQP solved. *I*_*m *_is updated and *RE*_*m *_becomes *RE*_*m*_|Δ. If the updated *RE*_*m *_is still greater than $MAX_{m}^{r}$, a further set of nodes of size *N_R *is released, otherwise all remaining nodes are released. The reduced MIQP is solved once again and *I*_*m *_and *RE*_*m *_updated accordingly. This is repeated until all nodes in the module have been released at one point and the procedure moves on to the next module. In order to determine the set of *N_R *nodes to be released in modules, we use a simple rule by first sorting nodes with non-decreasing indices and then assigning higher priority to nodes with smaller indices.

The above scheme is applied to all modules detected during Stage 1, with the sequential order of the modules maintained throughout the whole procedure. Future research can investigate the effect of changing the sequence of module reallocation, the appropriate selection of *MAX*_*m*_^*r *^and node prioritisation. Figure 1 illustrates the entire module detection strategy, iMod, encompassing Stages 1 and 2 of the mathematical programming algorithm reported above.

### Procedure to address resolution limitations

Although the modularity metric has been widely accepted as a standard measure to quantify the community composition in networks and detect modules, resolution limit problems can hinder its application. Such effects entail the failure of modularity optimisation to detect modules smaller than a scale which depends on the size of the network and the degree of inter-connectedness of the modules, as the algorithm tends to merge small modules to achieve larger modularity values [33,37,38]. Methodologies that aim to overcome resolution limits can provide deeper insights into finer structures of modules in complex networks and a more accurate depiction of community structure on the basis of the modularity measure.

In this section, we report a solution procedure (ResMod) that allows smaller modules that may not be detected in the initial modularity optimisation to become apparent. First, the two-stage approach for module detection, iMod, is applied to the whole network to obtain a partition into several modules. In order to determine if these modules comprise smaller modules, each module is considered as a disjoint subnetwork, ignoring links with other modules, and iMod is then applied once to each subnetwork.

The partition of the subnetwork into smaller modules is accepted as part of the community structure of the original network if its modularity as a disconnected entity (i.e. only considering the links involved in the subnetwork) is greater than an enforced threshold. If the partition of the subnetwork yields a value less than this threshold, the new decomposition is not accepted and the subnetwork remains intact as a community of the original network.

Here, a threshold of value of 0.3 is adopted as a representative community structure indicator, in accordance to previous reports [25,26,33,34]. This criterion is implemented to avoid over-partitioning that may hinder method applicability. We should note here that this implementation of a single and unvarying threshold to determine whether partitioning is required may not be enough to capture cases where random graphs (or partitions obtained by chance) have a modularity higher than 0.3. Ideally, this criterion should be complemented with an estimate of the statistical significance of the modularity achieved (see [4,34]) to ensure that this value is above a fluctuation margin. However, in practice, even this coarse-grained approach to resolution limitation problems seems to work well in proposing finer community structures for common complex networks and it is a good first step into research for improving modularity maximisation methods.

## Results and Discussion

The application of iMod to detect modules and ResMod to correct for potential resolution limitations is illustrated in this section through a number of real network examples. All implementations were performed in GAMS (General Algebraic Modeling System) [39] and mathematical models (MINLP and MIQP) are solved using SBB [40] and CPLEX [41] mixed integer optimisation solvers with computational limit of 3600 seconds, where necessary. Each round of a module detection experiment involves running iMod ten times and reporting the best and median modularity values (Table 1). ResMod is subsequently used on the partitioned networks to resolve resolution and identify finer modular structures that may be present. A comprehensive comparison of our approach to other module detection methodologies was performed and is discussed below to illustrate significant improvements over previous approaches.

**Table 1:** Computational results comparing the performance of modularity optimisation methodologies across several network examples.

Networks			iMod			EB	EIG	**C**_**3**_**/C**_**4**_	EO	SA	QCUT	Greedy
**Name**	***N***	***L***	**Median *Q***	**Best *Q***	***M***	***Q***						

Zachary	34	78	0.420	**0.420**	4	0.401	0.419	0.417	0.419		0.420	0.419
Dolphin	62	159	0.529	**0.529**	5	0.520					0.518	0.519
Les Miserables	77	254	0.560	**0.560**	6	0.540					0.560	0.556
P53	104	226	0.535	**0.535**	7						0.522	0.531
Jazz	198	2742	0.445	**0.445**	4	0.405	0.442	0.441	0.445		0.445	0.443
*E. coli*	418	519	0.780	**0.781**	19		0.766			0.752	0.776	0.779
*S. cerevisiae*	688	1079	0.768	**0.775**	25		0.759			0.740	0.766	0.764
*C. elegans*	453	2025	0.451	**0.453**	9	0.403	0.435	0.422	0.434		0.433	0.441
Email	1133	5451	0.575	**0.580**	9	0.532	0.572	0.567	0.574		0.576	0.543

Best modularity achieved across all methodologies and network examples is denoted in bold.

References: EB [25], EIG [9], C_3_/C_4 _[51] EO [32], SA [33], QCUT [34], Greedy [52]

A number of networks identified from the literature serve as test cases to showcase the efficiency of the computational methodology. Table 1 summarises all networks considered, their sizes and indicative results of the methodologies tested. Overall, nine examples were used with varying sizes, in terms of total number of nodes and links. These cases are inspired from social or biological relationships and represent well-studied cases in network analysis and related algorithm development.

**Figure 2:**
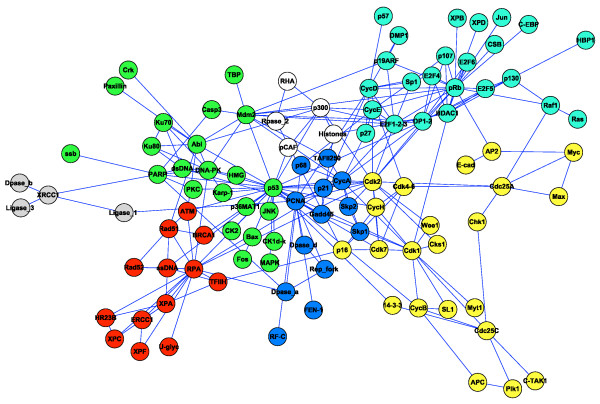
**Network representation of the p53 protein interactions**. Modules, as detected through iMod, are indicated by colour.

Networks describing social interactions in our study are (in ascending number of nodes): the Zachary network of social relationships in an American university club [42], the communications among dolphins constructed through a field study [43,44], relations among roles in the novel Les Miserables [45], a network of jazz musicians as described through their recordings [46] and a university network of email communication [47]. Biological networks assessed are: the p53 protein interaction network [11], the transcriptional network of the bacterium *Escherichia coli *[48], the transcriptional network of the yeast *Saccharomyces cerevisiae *[49] and the network of metabolic reactions of the nematode *Caenorhabditis elegans *[50]. Figure 2 shows the network representation of p53 protein interactions, with colours indicating modules as detected by iMod.

Our methodologies for community detection and resolution limitations are compared against the most widely used approaches that employ modularity maximisation. Here, a brief account of such previously developed algorithms is given, together with reference to the original publications for more details on the main properties of each algorithm.

An algorithm based on edge-betweenness (EB) [25] that involves the iterative removal of edges with the highest *betweenness *score to split the network into communities has been one of the very first attempts to use modularity maximisation for module detection. The eigenvector approach (EIG) was later proposed by the same group, where network modularity was rewritten as eigenvectors of a modularity matrix and lead to a spectral algorithm for community detection [9]. Edge-betweenness has recently been extended through the use of edge weights defined by the edge-clustering coefficient (C_3_/C_4_) to improve module detections [51]. Popular optimisation methodologies have been proposed as efficient means to achieve modularity maximisation, namely extremal optimisation (EO) [32] and simulated annealing (SA) [33]. Recently, heuristic algorithms have been proposed, i.e. one that relies on spectral graph partitioning and local search (QCUT) [34] and a greedy method for iterative grouping of nodes into communities (Greedy) [52]. Both of these methods show good performance compared to previous approaches. For Greedy and QCUT, we used the relevant software to evaluate modules and estimate the resulting modularity. For all other methods, the reported results are taken from the relevant published papers.

Extensive comparisons of performance across all above methodologies show that iMod achieved network partitions with the highest modularity (Table 1). Consistently better performance was noted for iMod throughout all examples studied. It is important to mention that even small improvements in modularity can differentiate between good and exceptional methods, as has been noted previously [9].

For the example of the p53 network (Figure 2), modules were mapped onto KEGG pathways and pathway enrichment was calculated against the human genome through SubpathwayMiner [53]. We compared enriched pathways for the iMod and Greedy partitions and, even for small differences in community structures detected, more pathways were significantly enriched in the iMod partition. Even though clearly more work is need along these lines, this is an early indication that module detection through iMod may be more meaningful biologically.

Comparative analyses are hindered to some extent by missing values in Table 1, as different network examples were assessed through each of the reported methodologies. For instance, the p53 example has been implemented in three methods (iMod, QCUT and Greedy), the Dolphin and Les Miserables networks were considered by four methodologies (iMod, EB, QCUT and Greedy), the *E. coli *and *S. cerevisiae *by five (iMod, EIG, SA, QCUT and Greedy) and the remaining four networks have been tested by different combinations of seven methodologies out of eight community detection algorithms considered in total. Such missing values indicate an impediment in related comparison efforts and it is suggested that the definition of network examples as standards, where algorithm development and evaluation can be benchmarked, is needed in order to facilitate and improve comparative analyses [54]. However, it should also be noted that this is one of the most comprehensive comparisons of module detection methodologies employing modularity maximisation, to our knowledge.

Benchmarking was also extended to simulated networks to illustrate the efficiency of iMod. A large number of artificial networks with known community structure was generated, as described previously [25]. These synthetic networks comprise 128 nodes and are partitioned into four communities of 32 nodes with degree equal to 16. In addition, we considered the case where degree was set equal to 5, as this represented a more realistic estimate of the average node degree in real networks (see Table 1). The mixing parameter, * μ*, i.e. the fraction of all links in a particular module that end outside this module, was varied from 0.1 to 0.5. Increasing the mixing parameter makes the modules of the 'true' community structure less well defined and the communities less easily detected. Testing for a mixing parameter greater than 0.5 was not deemed necessary, as it would contradict the definition of community structure, where more intra-community links than inter-community links should exist.

**Figure 3:**
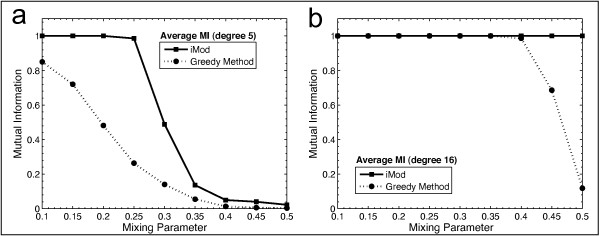
**Benchmarking of module detection performance with iMod and the Greedy algorithm**. Synthetic network examples (128 nodes, 4 modules) were generated with node degrees of 5 and 16 in (a) and (b) respectively. For each mixing parameter, μ, 100 networks were assessed. The agreement of modules detected with the known community structure was expressed via the mutual information measure. Consistently better performance was noted for iMod in all examples tested.

We tested how well iMod extracted this known structure and compared this to the Greedy algorithm [52], which was the next best performing method from the comparison reported in Table 1. The mutual information measure [55] was used to illustrate the agreement between the known and detected community structures, i.e. mutual information ranges from 0 (for dissimilar) to 1 for identical community structures. We generated 100 synthetic networks for each mixing parameter examined, each of these was analysed with iMod and the Greedy method, and the average mutual information was calculated. Figures 3a and 3b report the mutual information plotted against the mixing parameter for the synthetic networks to illustrate how close these methods were in revealing the known community structure.

Overall, iMod performed better for all examples tested. For node degree equal to 16, iMod and the Greedy method manage to retrieve the exact partition for all values of * μ *up to 0.35. Thereafter, iMod outperforms the Greedy method by continuing to extract the exact partition whereas the Greedy method's performance declines rapidly. In the case of degree equal to 5, iMod still achieves higher similarity to the known structure than the Greedy method for all values of * μ*.

### Detection of resolution limitations

Improved community structures are not achieved solely through maximisation of modularity; further refinement by addressing resolution limits of modularity maximisation is critically important. Network modules obtained with the iMod algorithm were further partitioned as described in the ResMod procedure, ignoring all inter-module links, as outlined above.

**Figure 4:**
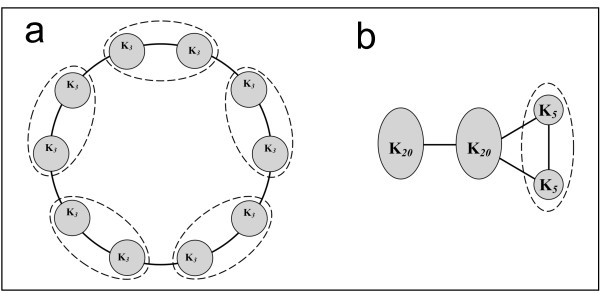
**Benchmarking of the procedure to address resolution limitations**. (a) Ring-shaped network, each circle denotes identical complete graphs (subgraphs of three nodes, K_3_). Subgraphs are connected with the minimum number of edges, as shown. Dotted lines indicate modules detected through modularity maximisation without correcting for resolution (iMod). After accounting for resolution limitations (ResMod), each complete graph is identified as a separate module, revealing the correct community structure of ten modules. (b) Y-shaped network comprising of complete subgraphs with twenty and five nodes (K_20_, K_5 _respectively), linked as shown. Resolution limitations in modularity maximisation lead to merging the two smallest subgraphs, thus yielding three modules. The ResMod algorithm can correctly identify all four modules present.

To illustrate how the proposed methodology can be used to overcome resolution limitations, two synthetic examples from the literature [33] are used, as they represent particularly challenging cases in module detection. These network examples are shown in figures 4a and 4b and summarised in Table 2. Both synthetic examples are rather extreme cases in terms of their topological properties and serve to verify the accurate detection of community structure where resolution limitations may pose significant problems. These are discussed in detail below.

**Table 2:** Computational results for modularity optimisation and resolution limits in simulated network examples.

Networks			iMod			ResMod	
**Name**	***N***	***L***	**Median *Q***	**Best *Q***	***M***	***Q_Reso***	***M***

Ring	30	40	0.6750	0.6750	5	0.6500	10
Y-shape	50	40	0.5426	0.5426	3	0.5416	4

Median and best modularity values (Q) are reported after module detection with iMod (out of ten runs) and after accounting for resolution problems with ResMod (Q_Reso).

The first example is a ring-shaped network composed of 10 identical complete graphs of three nodes each, represented by circles inter-connected by the minimal number of links (Figure 4a). This graph is an example of maximal modularity, since modularity converges to one as the number of complete graphs reaches infinity [33,56]. Modularity maximisation using iMod initially suggests the existence of 5 modules, in accordance to other approaches [33]. Through implementation of ResMod to correct for resolution limits by optimising each of the five communities further without considering the inter-module links, the two smaller groups within each module become apparent and the total number of modules is correctly identified as ten.

The second synthetic example comprises four groups of nodes (*y-shaped*, Figure 4b). Each group, denoted by a circle, consists of completely connected graphs: the two leftmost groups comprise 20 nodes and the two on the right consist of 5 nodes each [33]. Methods that perform modularity maximisation tend to merge the two smallest groups to yield the highest possible modularity value at the cost of an inaccurate detection of underlying community structure. Partitioning the network through iMod and optimising each module through ResMod yields the accurate number of four modules and the network is partitioned correctly.

In real networks, improved module structures have been detected for the dolphin, p53, *E. coli *and *S. cerevisiae *networks, while no improvement has been detected for the remaining examples. Excluding the dolphin network, where a marginal increase to the number of modules was observed after ResMod, all larger size networks have shown a significant increment to the number of modules proposed after the treatment for resolution. As indicated in Table 3, the number of modules more than doubled in the p53 and yeast networks and the same quantity was four-fold higher in the *E. coli *and *C. elegans *cases. Such wide differences clearly confirm that accurate module detection is particularly challenging in large networks where resolution problems are more pronounced.

**Table 3:** Computational results for module detection without correction for resolution problems (iMod) and after accounting for resolution (ResMod).

Network	iMod		ResMod	
**Name**	***Q***	***M***	***Q***	***M***

Zachary	0.420	4	0.420	4
Dolphin	0.529	5	0.504	7
Les Miserables	0.560	6	0.560	6
P53	0.535	7	0.469	16
Jazz	0.445	4	0.445	4
*E. coli*	0.781	19	0.675	79
*S. cerevisiae*	0.775	25	0.693	66
*C. elegans*	0.453	9	0.366	44
Email	0.580	9	0.432	72

Another computational methodology that accounts for resolution limitations is the use of simulated annealing (SA) for modularity maximisation, where simulated annealing is applied to each detected module to find out whether any sub-modules can be identified [33]. In comparison, the *E. coli *network was partitioned into 79 modules with a modularity of 0.675 with ResMod, compared to 76 modules with modularity of 0.661 in SA. For the yeast network, ResMod achieves 66 modules with modularity of 0.693, as opposed to 57 modules with a total community modularity of 0.677 in SA. In both cases, ResMod succeeded in further dividing a higher number of modules while still achieving partitions with better overall modularity scores. Furthermore, another possible advantage of the methodology presented here is an explicit account to avoid over-partitioning, through implementation of the threshold value. However, further work is planned in the future to address: (i) quality control measures in module discovery to assess whether the detected community structure reflects phenotypic properties well, and (ii) further development of measures to avoid over-partitioning when accounting for resolution problems.

## Conclusions

Community structure identification through modularity maximisation is hindered by (i) the NP-hard properties of the related optimisation problem and (ii) the resolution limitations introduced through the modularity measure. We have previously reported the detection of community structure in small to medium networks through a mixed integer quadratic programming procedure that guarantees global optimal solutions for modularity maximisation [30]. Here, we extend this work to tackle large size networks through an iterative optimisation procedure that performs well as evidenced through comparative analyses. As a further improvement, we also report methodological details of identifying and addressing resolution limitations, thus retrieving a more accurate representation of community structure from data.

Despite significant advances in the area of module detection through modularity optimisation, it is important to mention some caveats. First, modularity may not be the most appropriate measure of topological network features, as it can introduce limitations in practical applications [33,57]. Alternative measures have been proposed [36] and will be studied in future work in terms of their ability to enhance module detection. It is worth noting that solution procedures presented here are generic and can be implemented with any mathematical expression of community presence other than modularity.

Furthermore, the use of coarse-grained topological features as a means to represent a complex network may not always be sufficient in delineating the intricate relationships and phenotypic properties of the system at hand. For example, in biological networks modularity is a phenomenon linked to a varying contribution of evolutionary inheritance of features, genome organisation properties and functional attributes [8]. Enriched network abstractions (e.g. edge weight and directionality), development of more accurate fitness functions (for example to capture cooperation effects [58]), as well as methodologies incorporating dynamic features can all contribute to future advances.

Network theory and related computational approaches have significantly enhanced our ability to offer deep insights into the principles governing complex systems. Analysis of protein interactions has shed light into mechanisms of disease [59-61], the association of genetic to phenotypic properties [7,62] and biological species [2]. In this respect, the role of an accurate computational procedure to reveal the relations between the structure and functions in complex systems is important. Methodologies that allow communities to be detected both optimally and unambiguously, such as the ones presented in this paper, can greatly assist in this direction.

## Competing interests

The authors declare that they have no competing interests.

## Authors' contributions

GX, LGP and ST designed research, GX and LB performed research, GX, LB, LGP and ST analysed data, GX, LGP and ST wrote the paper.
